# Validation of a diagnostic prediction tool for colorectal cancer: a case–control replication study

**DOI:** 10.1093/fampra/cmac147

**Published:** 2023-01-05

**Authors:** Elinor Nemlander, Andreas Rosenblad, Eliya Abedi, Jan Hasselström, Annika Sjövall, Axel C Carlsson, Marcela Ewing

**Affiliations:** Department of Neurobiology, Care Sciences and Society, Division of Family Medicine and Primary Care, Karolinska Institutet, Solna, Sweden; Academic Primary Health Care Centre, Region Stockholm, Stockholm, Sweden; Department of Neurobiology, Care Sciences and Society, Division of Family Medicine and Primary Care, Karolinska Institutet, Solna, Sweden; Regional Cancer Centre Stockholm-Gotland, Region Stockholm, Stockholm, Sweden; Department of Medical Sciences, Division of Clinical Diabetology and Metabolism, Uppsala University, Uppsala, Sweden; Department of Neurobiology, Care Sciences and Society, Division of Family Medicine and Primary Care, Karolinska Institutet, Solna, Sweden; Academic Primary Health Care Centre, Region Stockholm, Stockholm, Sweden; Department of Neurobiology, Care Sciences and Society, Division of Family Medicine and Primary Care, Karolinska Institutet, Solna, Sweden; Academic Primary Health Care Centre, Region Stockholm, Stockholm, Sweden; Division of Coloproctology, Department of Pelvic Cancer, Karolinska University Hospital, Department of Molecular Medicine and Surgery, Karolinska Institutet, Stockholm, Sweden; Department of Neurobiology, Care Sciences and Society, Division of Family Medicine and Primary Care, Karolinska Institutet, Solna, Sweden; Academic Primary Health Care Centre, Region Stockholm, Stockholm, Sweden; Institute of Medicine, Department of Community Medicine and Public Health, Sahlgrenska Academy, University of Gothenburg, Gothenburg, Sweden

**Keywords:** colorectal cancer, diagnosis, general practice, replication, risk assessment tool, Sweden

## Abstract

**Background:**

Early detection of colorectal cancer (CRC) is crucial for survival. Primary care, the first point of contact in most cases, needs supportive risk assessment tools. We aimed to replicate the Swedish Colorectal Cancer Risk Assessment Tool (SCCRAT) for non-metastatic CRC in primary care and examine if risk factor patterns depend on sex and age.

**Methods:**

2,920 adults diagnosed with non-metastatic CRC during the years 2015–2019 after having visited a general practitioner the year before the diagnosis were selected from the Swedish Cancer Register and matched with 11,628 controls, using the same inclusion criteria except for the CRC diagnosis. Diagnostic codes from primary care consultations were collected from a regional health care database. Positive predictive values (PPVs) were estimated for the same 5 symptoms and combinations thereof as in the baseline study.

**Results:**

The results for patients aged ≥50 years old in the present study were consistent with the results of the SCCRAT study. All symptoms and combinations thereof with a PPV >5% in the present study had a PPV >5% in the baseline study. The combination of bleeding with abdominal pain (PPV 9.9%) and bleeding with change in bowel habit (PPV 7.8%) were the highest observed PPVs in both studies. Similar risk patterns were seen for all ages and when men and women were studied separately.

**Conclusion:**

This external validation of the SCCRAT for non-metastatic CRC in primary care replicated the baseline study successfully and identified patients at high risk for CRC.

**Key messages**

- Colorectal cancer identified at an early stage is important for the prognosis.
- Validated diagnostic prediction tools are warranted.
- A risk assessment tool for non-metastatic colorectal cancer has now been validated.

## Introduction

Colorectal cancer (CRC) is the third most common cancer worldwide and the second leading cause of cancer death.^1^ Europe has approximately half a million cases yearly and a quarter of a million deaths.^2^

Early detection of CRC is crucial for the patient’s prognosis. Even though Sweden has high survival rates, CRC still has a poor prognosis when discovered at an advanced stage: a 15% 5-year-relative survival rate when diagnosed in stage IV compared with 95%, 89%, and 68% when diagnosed in stages I, II, and III, respectively.^3^

Most cancer investigations start in primary health care (PHC) due to symptoms and signs. Like the United Kingdom, several European countries have implemented urgent referral pathways for suspected cancer. In 2016, urgent referrals for suspected CRC (standardized cancer patient pathways) were introduced in Sweden.^4^

The standardized cancer patient pathways are not designed to estimate the individual patient’s cancer risk but contain symptoms that should lead to the suspicion of cancer. This is challenging for general practitioners (GPs), who must assess the likelihood of CRC and prioritize investigations and treatments among large groups of patients seeking care with often focal, unspecific and/or common symptoms, each with a possible link to cancer. Diagnostic prediction tools (DPTs) for cancer, i.e. tools that translate epidemiological risk markers to applicable individual patient assessments, are lacking in Swedish PHC. Several types of cancer diagnostic tools for CRC based on patients’ symptom presentation have been developed in Europe, primarily in the United Kingdom.^5–10^ There is, however, insufficient evidence to suggest that cancer DPTs in PHC affect the clinical outcome^11,12^ and no cancer DPT has yet been included in UK guidelines.^13^ To create a solid evidence-base for DPTs for early detection of CRC in PHC, patient populations in many health care systems need to be studied, and the different DPTs externally validated to a higher extent^14^ due to variations in health care system organizations, cancer-related risk factors, symptom presentation, and documentation traditions. This should include exploratory as well as prospective confirmative studies.

In 2016 Ewing et al.^15^ published a case–control study based on regional health care databases from the southwestern Swedish Region Västra Götaland (1.7 million inhabitants) and the National Swedish Cancer Register (SCR), resulting in a Swedish CRC Risk Assessment Tool (SCCRAT) for non-metastatic CRC in individuals aged ≥50 years old for use in PHC.

The aim of the present study was to validate the SCCRAT on patients with CRC by replicating the already developed risk algorithm in a population in a different region, Region Stockholm, Sweden as well as examine if the risk marker patterns diverge depending on sex and age. We also aimed to compare the results from Region Stockholm with Region Västra Götaland.

## Materials and methods

### Study design and setting

This population-based matched case–control study utilized the Stockholm regional health care administration database (VAL)^16^ for identifying the entire population of Region Stockholm, comprising the Swedish capital Stockholm and surrounding areas (2.4 million inhabitants), and the SCR for identifying cases. All medical data in Region Stockholm, from public and private health care providers, are automatically transferred to the VAL database, which is used for health care planning, practice remuneration, and quality assessment.^16^

The SCR, established in 1958, is one of the oldest disease registers in Sweden and has high validity.^17^ All physicians, including pathology laboratories, in Sweden are obliged by law to report all incident cases of cancer from patients to the SCR.

### Study population and data sources

The selection of cases and controls emulated the process of the baseline study by Ewing et al.^15^ as closely as possible. Inclusion criteria for cases were:

- Diagnosed in Region Stockholm with CRC (ICD-10 codes C18–20) stages I–III during the years 2015–2019 (identified in the SCR),
- Alive and aged ≥18 years old at the date of the CRC cancer diagnosis (index date), and
- Having visited a GP in Region Stockholm during the year before the index date.

Exclusion criteria were:

- No controls available, or
- Having a previous cancer diagnosis (except non-melanocytic skin cancer) registered in the SCR during the 20 years before the index date.

The latter group was deliberately omitted to avoid GP consultations being a control of or concern over a previous cancer diagnosis. Controls were selected from the VAL database based on the index date of the matched case (date of cancer diagnosis of the case), using the same inclusion criteria as for the cases, except for the CRC diagnosis. For each case, up to 4 controls were matched on age, sex, and PHC unit visited, resulting in a total study population of 2,920 cases and 11,628 controls, with 2,890 (99.0%) of the cases having 4 controls. Data on GP visits during the year before the index date were obtained from the VAL database.

### The Swedish CRC Risk Assessment Tool

The SCCRAT is based on 5 symptoms and signs, identified by ICD-10 codes^18^ and KSH97-P codes (an abbreviated version of ICD-10 adapted to Swedish PHC).^19^ The KSH97-P coding system was, however, not used in the VAL database during the years of the present study and our study is thus based on ICD-10 coding. Table 1 gives the ICD-10 codes for the SCCRAT used in the present study.

**Table 1.** ICD-10 codes for symptoms included in the SCCRAT.

Change in bowel habit	Bleeding	Weight loss	Abdominal pain	Anaemia
K590	K625	R630	R100	D500
K591	K921	R634	R101	D508
R194	K922	R636	R102	D509
	R589	R638	R103	D641
			R104	D648
				D649

ICD-10, International Statistical Classification of Diseases and Related Health Problems, 10th revision.

The symptoms and signs are: *change in bowel habit*, *(rectal) bleeding*, *weight loss*, *abdominal pain*, and *anaemia*. These 5 symptoms were a result of a merging process of all collected ICD-10 codes, after which the codes underwent univariable conditional regression. Variables associated with cancer entered multivariable analyses, after which a list of statistically significant variables associated with CRC was compiled and a positive predictive value (PPV) was calculated for each variable.^15^

For each symptom and combination of 2 symptoms, SCCRAT gives the PPVs for CRC, under an assumed prevalence of 0.25% for the disease in the studied group, when having at least 1 GP visit with the symptom in question, at least 2 different GP visits with the same symptom, or 2 different symptoms at 1 or more GP visits. For reasons of comparability, this assumed prevalence is the same as in the baseline study by Ewing et al.^15^

### Statistical analyses

Categorical data are reported as frequencies and percentages, *n* (%), while discrete and continuous data are given as means with accompanying SDs. The associations between the 5 symptoms (predictors) and the outcome CRC diagnosis (yes/no) were calculated using adjusted and unadjusted conditional logistic regression analysis, with the adjusted analyses including all 5 risk factors simultaneously. The results are presented as odds ratios with accompanying 95% confidence intervals (CIs). SCCRAT is evaluated by calculating PPVs with accompanying 95% CIs for each combination of symptoms in the model,^20^ separately for all ages, individuals aged ≥50 and ≥70 years old, respectively, as well as men and women regardless of age. PPVs are presented as percentages (%). All statistical analyses were performed in R 4.1.3 (R Foundation for Statistical Computing, Vienna, Austria) with 2-sided *P* values <0.05 considered statistically significant.

## Results

Characteristics of the 2,920 CRC cases and 11,628 matched controls included in the present study are given in Table 2. Of the cases 1,343 (46%) had a stage III CRC, 1,483 (50.8%) were males, and 1,602 (54.9%) were between 50 and 70 years old at the date of the CRC diagnosis, with a mean (SD) age of 70.7 (12.6) years. During the year before the index date, 1,457 (49.9%) of the cases had visited a GP at least once with at least 1 of the 5 symptoms. Among the controls, the corresponding number was 906 (7.8%). The present validation is a replication of a previous baseline study and includes almost 6 times as many patients as the baseline study in Region Västra Götaland.

**Table 2.** Characteristics of CRC cases and matched controls visiting a GP during the year before the index date.

Variable	Cases	Controls	*P* value^a^
	*n* = 2,920	*n* = 11,628	
TNM stage			
I	731 (25.0)	–	–
II	846 (29.0)	–	–
III	1,343 (46.0)	–	–
Men, *n* (%)	1,483 (50.8)	5,901 (50.7)	–
Age at index date (years), mean (SD)	70.7 (12.6)	70.6 (12.5)	–
<50 years, *n* (%)	211 (7.2)	846 (7.3)	–
≥50 to <70 years, *n* (%)	1,602 (54.9)	6,333 (54.5)	–
≥70 years, *n* (%)	1,107 (37.9)	4,449 (38.3)	–
Time from first GP visit to index date (days), mean (SD)	232 (118)	262 (96)	<0.001
Total number of GP visits, mean (SD)	5.3 (6.0)	4.6 (5.4)	<0.001
Number of GP visits with at least 1 symptom			
At least 1 visit, *n* (%)	1,457 (49.9)	906 (7.8)	<0.001
At least 2 visits, *n* (%)	485 (16.6)	286 (2.5)	<0.001
Number of GP visits with change in bowel habit			
At least 1 visit, *n* (%)	439 (15.0)	249 (2.1)	<0.001
At least 2 visits, *n* (%)	101 (3.5)	56 (0.5)	<0.001
Number of GP visits with bleeding			
At least 1 visit, *n* (%)	220 (7.5)	47 (0.4)	<0.001
At least 2 visits, *n* (%)	34 (1.2)	6 (0.1)	<0.001
Number of GP visits with weight loss			
At least 1 visit, *n* (%)	28 (1.0)	19 (0.2)	<0.001
At least 2 visits, *n* (%)	8 (0.3)	5 (0.04)	0.001
Number of GP visits with abdominal pain			
At least 1 visit, *n* (%)	449 (15.4)	405 (3.5)	<0.001
At least 2 visits, *n* (%)	126 (4.3)	97 (0.8)	<0.001
Number of GP visits with anaemia			
At least 1 visit, *n* (%)	528 (18.1)	241 (2.1)	<0.001
At least 2 visits, *n* (%)	184 (6.3)	110 (0.9)	<0.001
Total number of different symptoms observed			<0.001
None, *n* (%)	1,463 (50.1)	10,722 (92.2)	
One, *n* (%)	1,265 (43.3)	852 (7.3)	
Two, *n* (%)	178 (6.1)	53 (0.5)	
Three, *n* (%)	13 (0.4)	1 (0.009)	
Four, *n* (%)	1 (0.03)	0 (0.0)	

Only GP visits occurring during the year before the index date are included. Symptoms considered were: change in bowel habit, bleeding, weight loss, abdominal pain, and anaemia. Index date is defined as the date of CRC diagnosis for the matched case patient.

^a^ *P* values from unadjusted conditional logistic regression analyses for the outcome non-metastatic CRC stages I–III.

For all symptoms, GP visits were more common among cases than controls. *Anaemia* was the most common symptom, which had been observed at a GP visit for 528 (18.1%) of the cases, compared with only 241 (2.1%) of the controls, while *weight loss* was least common, observed among only 28 (1.0%) of the cases and 19 (0.2%) of the controls. The observed differences were in all cases statistically significant (all *P* values ≤0.001, Table 2).

### Conditional logistic regression analyses

Results from adjusted and unadjusted conditional logistic regression analyses are given in Table 3. All symptoms were significantly associated with the outcome CRC stages I–III, in both adjusted and unadjusted analyses (all *P* values <0.001). Notably, having had a GP visit with the symptom (rectal) *bleeding* was associated with a 27.5 times higher risk of being diagnosed with CRC stages I–III in the adjusted analysis, while *anaemia* was associated with a 12.7 times higher risk.

**Table 3.** Results from adjusted and unadjusted conditional logistic regression analyses for the outcome non-metastatic CRC stages I–III in cases and controls matched on age and sex.

Symptom	Unadjusted		*P* value	Adjusted^a^		*P* value
	OR	95% CI		OR	95% CI	
Change in bowel habit	8.2	6.9–9.8	<0.001	9.3	7.7–11.3	<0.001
Bleeding	19.8	14.3–27.4	<0.001	27.5	19.3–39.2	<0.001
Weight loss	6.1	3.3–11.1	<0.001	3.6	1.7–7.5	<0.001
Abdominal pain	5.2	4.5–6.1	<0.001	5.9	5.0–7.0	<0.001
Anaemia	10.8	9.1–12.8	<0.001	12.7	10.5–15.3	<0.001

OR, odds ratio.

^a^Adjusted for all other variables in the table.

### Validation of the SCCRAT: individuals aged ≥50 years old

**Fig. 1.**
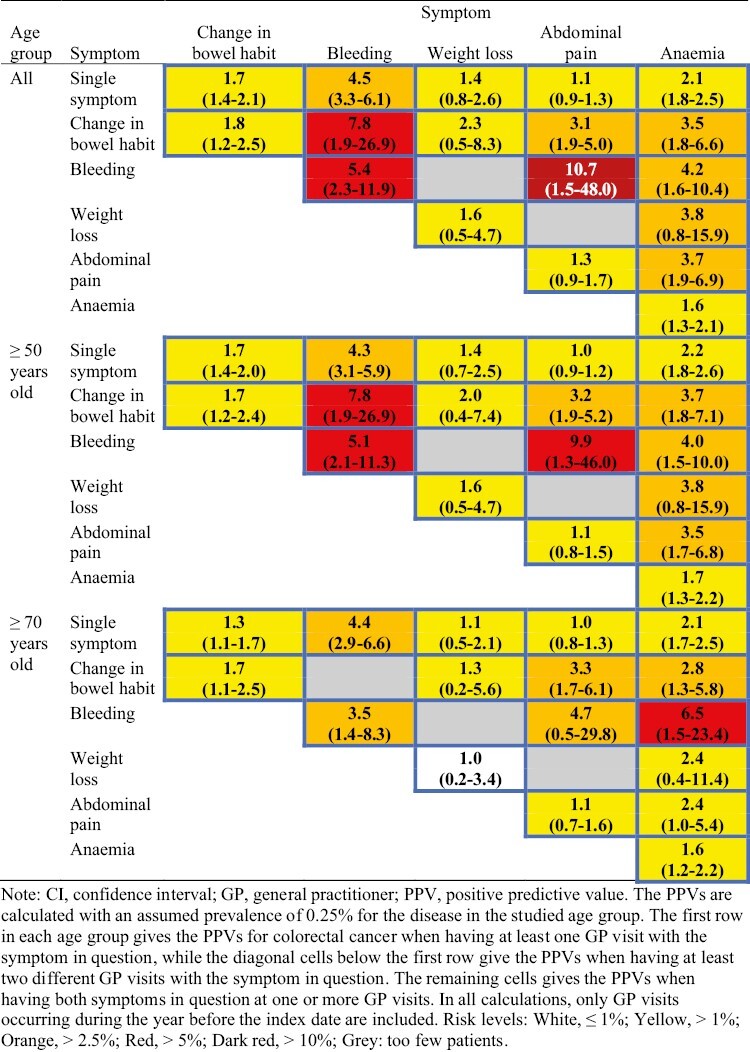
Swedish colorectal cancer risk assessment tool (SCCRAT) in the Stockholm Region for non-metastatic colorectal cancer stage I–III among patients according to age. Each cell gives the PPV (%) with 95% CI for the symptom(s) in question (for colour figure refer to online version).

Figure 1 gives the SCCRAT separately for all ages, individuals aged ≥50 years old and individuals aged ≥70 years old, respectively. Figure 1 shows the PPVs for individuals aged ≥50 years old and serves as a validation of the results in the baseline study.^15^ Notably, most of the observed PPVs in Fig. 1 are higher than the corresponding baseline PPVs, with only 5 PPVs being lower: The combination of *change in bowel habit* and *bleeding*, which with a PPV of 13.7% was the highest observed PPV in the baseline study, had a PPV of 7.8% in the present study. This value is, however, still the next highest PPV for this age group in Fig. 1. Likewise, the combination of *bleeding* and *abdominal pain*, with the next highest PPV of 12.2% in the baseline study, was 9.9% in the present study. This is, however, the highest PPV for this age group in the present study. Finally, having at least 2 different GP visits with *weight loss*, the combination of *weight loss* and *anaemia*, and the combination of *abdominal pain* and *anaemia*, with PPVs of 1.6%, 3.8%, and 3.5%, respectively, were slightly lower than the corresponding baseline PPVs of 2.9%, 5.6%, and 4.2%. Finally, it should be noted that all PPVs with a value >5% in the present study had a PPV >5% also in the baseline study. Likewise, all PPVs with a value >2.5% in the present study, except for the combination of *change in bowel habit* and *abdominal pain*, had a PPV >2.5% also in the baseline study.

### SCCRAT for other ages and stratified on sex

For the other age groups in Fig. 1, the differences compared with the group aged ≥50 years old are in general small. A notable difference is, however, that the PPV of 6.5% for the combination of *bleeding* and *anaemia* in the age group ≥70 years old is considerably higher than the corresponding PPVs of 4.2% for the group of all ages and 4.0% for the group aged ≥50 years old. In contrast, the PPV of 4.7% for the combination of *bleeding* and *abdominal pain* in the age group ≥70 years old is considerably lower than the corresponding PPVs of 10.7% for the group of all ages and 9.9% for the group aged ≥50 years old.

**Fig. 2.**
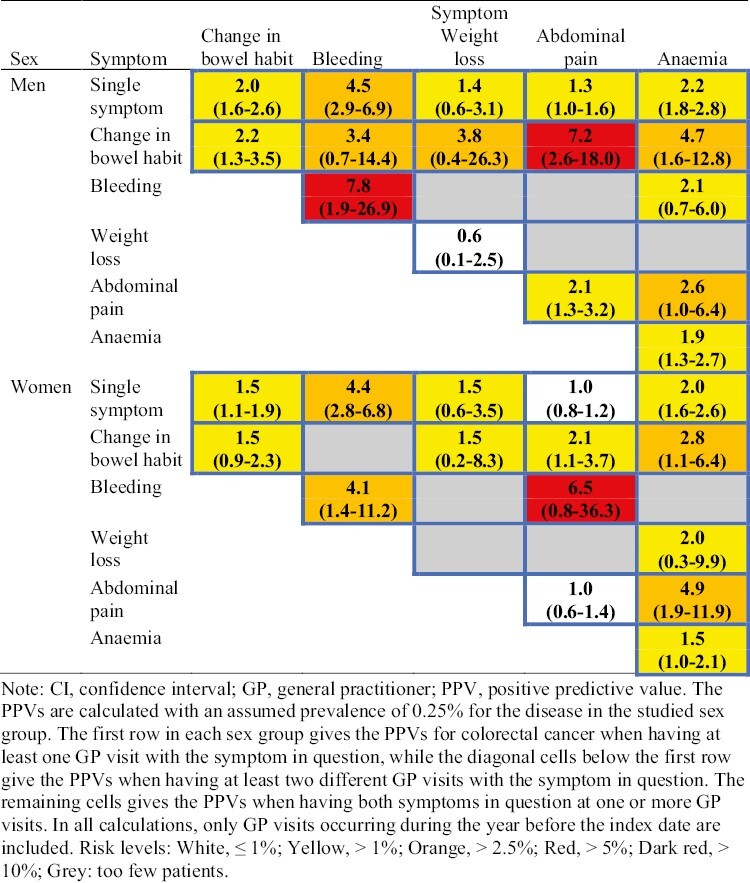
Swedish colorectal cancer risk assessment tool (SCCRAT) for non-metastatic colorectal cancer stage I–III among patients according to sex. Each cell gives the PPV (%) with 95% CI for the symptom(s) in question (for colour figure refer to online version).

Figure 2 gives the SCCRAT separately for men and women. Although the risk patterns overall are similar regardless of sex, a noticeably difference is the more than 3 times higher PPV for the combination of *change in bowel habit* and *abdominal pain* among men compared with women, 7.2% vs. 2.1%. Likewise, the PPV of 7.8% for having at least 2 different GP visits with *bleeding* observed for men is almost double the corresponding PPV of 4.1% for women.

## Discussion

The results of the present study are consistent with the results in the baseline study by Ewing et al.^15^ When we applied an earlier calculated risk algorithm based on 5 symptoms and signs to a different population, they were found to be independently associated with non-metastatic CRC, and several combinations of these had high predictive value for non-metastatic CRC. In particular, various combinations of change in *bowel habit*, *bleeding*, *anaemia*, and/or *abdominal pain* had high PPVs. Moreover, all PPVs with a value >5% in the present study had a PPV >5% also in the baseline study. Similar risk patterns were seen for all ages and when men and women were studied separately.

### Strengths and limitations

The main strength of the present study is that it is based on the entire population of a region with 2.4 million inhabitants and all models are based on a real-world health care situation, which provides the opportunity for generalizable conclusions in relation to early cancer detection. Both the present replication study and the baseline study in Region Västra Götaland had nearly no missing cases of CRC, as all subsidized health care was included. Although the populations showed small differences as regards age and cancer stage the risk algorithms constructed based on the Stockholm populations were very similar. The large sample size in the present study made it possible to reliably validate the results of the baseline study as well as to construct separate SCCRATs for different age groups and for both sexes.

The use of unique personal identification numbers enabled the linking of data on diagnoses registered in Region Stockholm with high-quality national data on all CRC diagnoses, with stage at diagnosis verified by pathologists from the SCR.

The use of ICD-10 codes can be considered both a strength and a limitation. The risk of both selection and recall bias is small as the information is collected prospectively on all patients. However, this presupposes that all presented symptoms have been registered correctly. It has been described in previous studies that GPs do not code for all symptom diagnoses presented in the consultation. Important information about symptoms can instead be stated, e.g. in free text in the medical record, information that may be lost in this study.^21^ To investigate whether the inclusion of symptoms and signs given in free text could affect the results of the SCCRAT would be of great value.

The SCCRAT is based on 5 symptoms and signs known to be associated with CRC. Other known factors of importance for CRC such as family history of CRC are not coded in PHC and could therefore not be included in the SCCRAT. Only patients who consulted their physician in the year before CRC diagnosis were included in this study, leaving out patients diagnosed after only consulting the emergency room or found through screening. However, most patients diagnosed with cancer present with symptoms in primary care, and it is the GP that initiates the diagnostic pathway of patients later diagnosed with cancer.^22–24^ Our way of validation was done with the presupposition that the SCCRAT was complete. If we had chosen a broader approach other diagnoses and symptoms may have turned up as predictors of cancer.

### Results in perspective

Although several other CRC DPTs, similar to SCCRAT, has been developed based on registry data,^14^ these have not yet been deemed fully useful.^12^ At this stage of development, it is important to investigate the reproducibility of DPTs in various health care systems. The Swedish and UK populations face the same medical issues such as an ageing population, lifestyle-related conditions, cancer, cardiovascular diseases, and comorbidity. Patients in Sweden consult their GP less often than patients in England but the consultation last longer, on average 20 min compared with 10 min in England.^25^ Both countries have primarily tax-based health systems. In Sweden, most GPs are publicly employed, whereas in the United Kingdom they are mostly self-employed.^26^

A qualitative study that investigated potential prediagnostic differences that could explain some of the differences in cancer survival between the United Kingdom and Sweden, did not observe any differences in how symptoms were experienced and in awareness of what the symptoms might mean.^27^ However, there were differences in patients’ willingness to visit and revisit their GP.^27^ This would suggest that DPT could be transferrable between United Kingdom and Sweden but should be tested in both countries first.

An important question is whether the tools can affect detection at earlier cancer stage at diagnosis. What distinguishes SCCRAT from those developed in the United Kingdom is that it shows combinations of symptoms and signs in patients with non-metastatic CRC, thus focussing on discovery of CRC in stages with better survival.

In the United Kingdom, the patient’s probability of having an undiagnosed cancer based on symptoms and signs are getting more attention. In the PHC guidelines for referral, the National Institute for Health and Care Excellence (NICE) threshold for urgent referral was set at a 3% risk of undiagnosed cancer 2015.^13^ A threshold for urgent referral has not been decided in Sweden but is needed if DPTs are to be implemented into clinical practise.

### Implications for research and clinical practice

Identifying CRC via symptoms and signs is the major clinical pathway for diagnosis, even in a setting with CRC screening programmes. Implementing a useful DPT could help GPs identify patients at high risk of non-metastatic CRC.

The research presented here indicates that the SCCRAT for non-metastatic CRC in individuals aged >50 years for use in PHC is valid also in Region Stockholm. It remains to investigate the SCCRAT prospectively and its effects on time to diagnosis, cancer stage at diagnosis, and other health-related outcomes in diagnosed patients.

## Conclusion

The present study is a successful external replication of a previously published risk assessment tool for non-metastatic CRC in PHC. Furthermore, we also identified patients at high risk with the use of a simple tool based on symptom registration. Tools were also created for different age groups and for men and women separately. We call for DPTs that are based on free text symptoms from PHC records that make use of the information at hand in the clinical situation in PHC.

## Supplementary Material

cmac147_suppl_Supplementary_Checklist

## Data Availability

Data are available upon reasonable request from the corresponding author. All diagnostic data upon which the study are based can be obtained from rc.datauttag@vgregion.se.
